# Analysis of genetic copy number changes in cervical disease progression

**DOI:** 10.1186/1471-2407-10-432

**Published:** 2010-08-16

**Authors:** Frank A Policht, Minghao Song, Svetlana Sitailo, Anna O'Hare, Raheela Ashfaq, Carolyn Y Muller, Larry E Morrison, Walter King, Irina A Sokolova

**Affiliations:** 1 Abbott Molecular Inc, 1300 E. Touhy Ave, Des Plaines, IL 60018 USA; 2 Caris Diagnostics, Irving, TX 75063 USA; 3 Dept. of Obstetrics and Gynecology, University of New Mexico Health Sciences Center, Albuquerque, NM 87131 USA; 4 GE Healthcare, Whitchurch, Cardiff, CF14 7YT, UK

## Abstract

**Background:**

Cervical dysplasia and tumorigenesis have been linked with numerous chromosomal aberrations. The goal of this study was to evaluate 35 genomic regions associated with cervical disease and to select those which were found to have the highest frequency of aberration for use as probes in fluorescent in-situ hybridization.

**Methods:**

The frequency of gains and losses using fluorescence in-situ hybridization were assessed in these 35 regions on 30 paraffin-embedded cervical biopsy specimens. Based on this assessment, 6 candidate fluorescently labeled probes (8q24, Xp22, 20q13, 3p14, 3q26, CEP15) were selected for additional testing on a set of 106 cervical biopsy specimens diagnosed as Normal, CIN1, CIN2, CIN3, and SCC. The data were analyzed on the basis of signal mean, % change of signal mean between histological categories, and % positivity.

**Results:**

The study revealed that the chromosomal regions with the highest frequency of copy number gains and highest combined sensitivity and specificity in high-grade cervical disease were 8q24 and 3q26. The cytological application of these two probes was then evaluated on 118 ThinPrep™ samples diagnosed as Normal, ASCUS, LSIL, HSIL and Cancer to determine utility as a tool for less invasive screening. Using gains of either 8q24 or 3q26 as a positivity criterion yielded specificity (Normal +LSIL+ASCUS) of 81.0% and sensitivity (HSIL+Cancer) of 92.3% based on a threshold of 4 positive cells.

**Conclusions:**

The application of a FISH assay comprised of chromosomal probes 8q24 and 3q26 to cervical cytology specimens confirms the positive correlation between increasing dysplasia and copy gains and shows promise as a marker in cervical disease progression.

## Background

Cervical cancer is one of the three major cancer types, along with breast and ovarian, specifically threatening women's health. Cervical cancer remains one of the most common cancers among women worldwide and the leading cause of death in many countries, especially in developing countries [1]. PAP smear cervical cytology screening reduced the incidence and death rate from cervical cancer by up to 80% [2]. Recently, infection with human papilloma virus (HPV) has been confirmed as the major etiological cause of cervical cancer [3]. ALTS (ASCUS/LSIL Triage Study) and several other major studies have been conducted to evaluate the usefulness of HPV testing for risk stratification of cervical cancer [4,5]. Despite significant progress, the molecular events that allow HPV infected cells to develop into cancer cells have not been completely elucidated [6]. Chromosomal instability at a numerical or structural level is a hallmark of malignant tumors. Deletion, duplication and amplification of various genomic regions have been demonstrated to be present in many types of cancers [7,8]. Fluorescent in-situ hybridization (FISH) represents a molecular technique that allows the detection of numerical and structural abnormalities in interphase cell nuclei. The goal of this study was to compare copy number variations in critical chromosomal regions in normal patients and in patients with varying severity of cervical dysplasia and cervical cancer. It was important to determine if chromosomal aberrations occur early in tumorigenesis and whether they can be used as the markers of cervical cancer cells. To achieve this goal we performed a multi-step selection of FISH probes comprised of 35 different genomic regions. We began with a literature-based analysis of chromosomal loci shown to be involved in the development of the cervical cancer. We then prepared and tested candidate probes on histological samples comprised of normal donors and patients with CIN1, CIN 2, CIN3 and cervical cancer. Chromosomal loci were compared based on copy number variations, percent positive cases in each cohort, sensitivity and specificity parameters. The results of histological evaluation indicated that 8q24 and 3q26 probes might be useful markers of cervical cancer progression. Therefore, 8q24 and 3q26 FISH probes were combined into one probe set and tested on a cohort of 118 cytological Thin-prep specimens to assess its utility.

## Methods

### Histological Specimens

For the initial evaluation of 35 probes, a collection of 30 de-identified slides with paraffin-embedded tissue sections from CHTN (Cooperative Human Tissue Network) were used for evaluation. All CHTN divisions have full reviews and approvals from their local IRB (Institutional Review Board). This group consisted of 10 CIN1 (cervical intraepithelial neoplasia), 10 CIN2/CIN3, and 10 SCC (squamous cell carcinoma) samples. For the subsequent detailed analysis with 6 probes, a cohort of 106 de-identified paraffin-embedded tissues which consisted of 21 Normal specimens, 19 CIN1 specimens, 27 CIN2 specimens, 20 CIN3 specimens, and 19 SCC specimens from the University of Texas Southwestern (UTSW) were utilized. All histological specimens consisted of ectocervical biopsies obtained during colposcopic examination and were approved by the IRB at UTSW. Histological specimens were subjected to a second review by a qualified pathologist (R. A.) for final diagnostic designation.

### Cytological Specimens

Cytological analysis was performed on 118 residual ThinPrep™ specimens which consisted of 19 Normal, 25 ASCUS (atypical squamous cells of undetermined significance), 35 LSIL (low-grade squamous intraepithelial lesion), 23 HSIL (high-grade squamous intraepithelial lesion), and 16 Cancer samples. Specimens were prepared according to the ThinPrep protocol. Samples were obtained from the UTSW and the Mayo Clinic and were approved for research by their respective Institutional Review Boards.

### Probes and Formulations

A total of 35 probes were used for initial evaluation. These 35 probes consisted of 13 centromeric probes (CEP^®^) for chromosomes 1, 6, 7, 8, 9, 10, 11, 12, 15, 16, 17, 18, and X, as well as 22 locus-specific identifier (LSI^®^) probes (1p31, 1q41, 2p24, 2q23, 2q33, 3p14, 3p21, 3q26, 4p15, 4p16, 5p13, 5p15, 6p21, 6q16, 7p12, 8q24, 11p15, 11p23, 11q13, 20q13, Xp22, and Xq11). Selection of probes was based on literature review that revealed frequent aberrations in cervical dysplasia and cancer [9-23]. LSI and CEP probes were obtained from Vysis/Abbott Molecular, Inc. (Des Plaines, IL). Based on the results of the initial evaluation, a subset of 6 probes (5 LSI, 1 CEP) was generated. CEP3 was included for the purpose of evaluating true amplification of arm versus polyploidy. The probes included for subsequent evaluation were combined into two probe sets. The first probe set consisted of 8q24 (SpectrumGold™), 20q13 (SpectrumRed™), Xp22 (SpectrumGreen™), and CEP15 (SpectrumAqua™). The second probe set included 3p14 (SpectrumGreen), 3q26 (SpectrumGold), and CEP3 (SpectrumAqua). For cytological analysis, the probe set consisted of 8q24 (SpectrumGold) and 3q26 (SpectrumGreen).

### Histological Sample Pretreatment and Hybridization

Histological specimens were pretreated three times in Hemo-De (Scientific Safety Solvents) for 5 minutes each at room temperature followed by two 1-minute rinses in 100% ethanol (EtOH) at room temperature, incubation in 45% formic acid/0.3% hydrogen peroxide for 15 minutes at room temperature and then rinsed in deionized water for 3 minutes. Slides were then incubated in pretreatment solution (Abbott Molecular, Inc.) at 80°C for 10 minutes, rinsed for 3 minutes in deionized water, incubated 5-10 minutes in proteinase K solution at 37°C, and rinsed again for 3 minutes in deionized water. Slides were dehydrated for 1 minute each in 70% EtOH, 85% EtOH, and 100% EtOH and then air dried. Ten microliters of each respective probe hybridization mix (LSI^® ^buffer, blocking DNA, labeled probes) were added to the specimens, a coverslip applied, and sealed with rubber cement. Slides were codenatured for 5 minutes at 72°C and hybridized for 16 hours at 37°C on a HYBrite (Vysis/Abbott Molecular, Inc.). Following hybridization, coverslips were removed and slides were washed in 2X SSC/0.3% NP-40 at 73°C for 2 minutes and subsequently in 2X SSC/0.1% NP-40 for 1 minute at room temperature. Ten microliters of DAPI I counterstain were placed on the slide and a coverslip applied.

### Cytological Sample Pretreatment and Hybridization

Cytological specimens were pretreated in 72°C 2X SSC for 2 minutes followed by a 10 minute incubation in pepsin solution (0.5 mg/ml in 10 mM HCl) at 37°C. Slides were then rinsed in 1X PBS for 5 minutes, fixed in a 1% neutral-buffered formalin solution for 5 minutes and rinsed again in 1X PBS for 5 minutes. Dehydration of slides was performed via an ethanol series: 1 minute each in 70% EtOH, 85% EtOH, and 100% EtOH, followed by air-drying. Ten microliters of the probe hybridization mix (LSI^® ^buffer, blocking DNA, labeled probes) were added to the specimens, slides were coverslipped, and sealed with rubber cement. Slides were codenatured for 2 minutes at 72°C and hybridized for 16 hours at 37°C on a HYBrite (Vysis/Abbott Molecular, Inc.). Following hybridization, coverslips were removed and slides were washed in 2X SSC/0.3% NP-40 at 73°C for 2 minutes and subsequently in 2X SSC/0.1% NP-40 for 1 minute at room temperature. Ten microliters of DAPI I counterstain were placed on the slide and a coverslip applied.

### FISH Evaluation - Histological Specimens

Following hybridization, slides were evaluated using fluorescence microscopy with SpectrumGold, SpectrumOrange™, SpectrumRed, SpectrumGreen, and SpectrumAqua fluorescence filters (Vysis/Abbott Molecular, Inc., Des Plaines, IL). Tissue orientation was identified and the epithelial layer of the tissue was enumerated. Cells were considered to be chromosomally abnormal if they possessed three or more signals. Enumeration was completed upon identification of 25 abnormal cells or complete analysis of the epithelial layer. Samples were considered positive if 4 or more chromosomally abnormal cells were found for each probe being evaluated.

### FISH Evaluation - Cytological Specimens

Following hybridization, slides were evaluated using fluorescence microscopy with SpectrumGold\and SpectrumRed fluorescence filters. Specimens were enumerated methodically, beginning with one corner of the cellular area, and enumerating each consecutive field of view from top to bottom and left to right. Cells were considered to be chromosomally abnormal if individual probes presented three or more signals. Enumeration was completed upon identification of 25 abnormal cells or the complete analysis of the cellular area. If 25 abnormal cells were recorded before completion of the entire area, the fraction of slide that was enumerated was recorded and the total number of abnormal cells per slide was calculated using this fraction.

### Statistical Analysis

Statistical analysis was performed using computer assisted correlation analysis with JMP Statistical Discovery Software (SAS; Cary, NC).

## Results

### Initial Probe Evaluation

The initial assessment of probes involved in cervical disease progression was executed on a limited set of samples (10 CIN1, 10 CIN2/3, 10 SCC specimens). The evaluation of the 35 potential probes was based on the frequency of aberrations observed for each histological category (CIN1, CIN2/3, SCC). An initial compilation and analysis of the enumeration data revealed that copy gains were significantly more prevalent than copy losses (Table 1); hence, we decided to focus our analysis mainly upon copy gains. In selecting the best candidate probes, we wanted to identify probes that revealed the highest frequency of gains in both the CIN2/3 and SCC categories, while maintaining a low frequency of copy gain detection in the CIN1 category. Using histology as the gold standard, the probes would exhibit copy number changes in 100% of cancer cases and 100% of CIN2/3 cases. Moreover, copy gains for the CIN1 category should be as close to 0% as possible. Based on this method of probe selection, the probes were initially sorted according to highest frequency of copy gains in cancer cases. For each probe we determined the percent of samples found positive based on our criteria (see methods) for groups of cancer and CIN2/3 cases (see Table 1). A threshold of 70% positive cases for categories of cancer and CIN2/3 was selected for exclusion of non-viable candidate probes. This threshold was determined to be an effective level of exclusion based on the limited size of the sample set. Of the 35 probes evaluated, 32 probes demonstrated copy gains in more than 70% of cancer samples whereas 12 probes were able to detect copy gains in at least 70% of CIN2/3 lesions.

**Table 1:** Percent probe copy gains and losses for each histological category (CIN1 - n = 10; CIN2/3 - n = 10; SCC - n = 10)

	% of Samples with Probe Copy Gains				% of Samples with Probe Copy Loss		
**Probe**	**CIN^a^1**	**CIN^a^2-3**	**SCC^b^**	**Probe**	**CIN^a^1**	**CIN^a^2-3**	**SCC^b^**

8q24	0	80	100	3p14.2	20	20	80

3q26	30	80	100	2q33-q34	11	11	20

5p13	30	80	100	Xq11-q12	22	0	20

5p15.2	40	80	100	3p21-p23	11	0	18

CEP^c^8	30	78	100	Xp22.3	20	0	11

CEP^c^X	50	75	100	1q41	0	0	10

CEP^c^10	18	70	100	7p12.3-p12.1	0	0	10

CEP^c^16	20	60	100	CEP^c^7	10	0	10

CEP^c^12	10	56	100	2q23-q37	0	10	0

CEP^c^6	10	50	100	6q16.3-q21.3	0	10	0

CEP^c^9	0	44	100	6p21.2	10	10	0

CEP^c^11	20	44	100	8q24.12-24.13	0	0	0

CEP^c^1	13	43	100	CEP^c^16	0	0	0

CEP^c^17	10	40	100	20q13.2-q13.3	0	0	0

CEP^c^7	30	60	91	CEP^c^15	0	0	0

20q13.2-q13.3	10	80	90	2p24.1	0	0	0

CEP^c^15	0	70	90	CEP^c^18	0	0	0

2p24.1	20	67	90	11p15.5	0	0	0

1q41	9	60	90	11q13	0	0	0

7p12.3-p12.1	10	60	90	1p31.1.1	0	0	0

Xq11-q12	11	60	90	4p16.3	0	0	0

CEP^c^18	11	60	90	11q23	0	0	0

11q13	27	60	90	4p15.3	0	0	0

11p15.5	18	50	90	3q26	10	0	0

11p23	45	30	90	5p13	10	0	0

6q16.3-q21.3	10	40	82	5p15.2	10	0	0

6p21.2	0	10	82	CEP^c^8	10	0	0

Xp22.3	0	70	80	CEP^c^X	10	0	0

1p31.1.1	9	70	80	CEP^c^12	10	0	0

4p15.3	20	50	80	CEP^c^11	10	0	0

4p16.3	30	50	80	CEP^c^9	10	0	0

2q23-q37	20	30	80	CEP^c^17	10	0	0

3p21-p23	14	86	55	CEP^c^6	10	0	0

2q33-q34	33	44	50	CEP^c^1	13	0	0

3p14.2	10	60	9	CEP^c^10	18	0	0

^a ^= cervical intraepithelial neoplasia

^b ^= squamous cell carcinoma

^c ^= centromeric probe

The final criterion for probe selection based on copy gains was a low percentage of copy gain detection in the CIN1 category in combination with highest percentages of copy gain detection for CIN2/3 and Cancer. This evaluation generated a set of 5 probes that detected copy gains in ≤ 10% of CIN1, and ≥ 70% of CIN2/3 and Cancer lesions: 8q24, 20q13, Xp22, 1p31, and CEP15 probes. Two of the probes - Xp22 and 1p31 - were nearly identical in overall performance across the 3 categories in this phase of our study. Based on the similarity in performance in high-grade/cancer lesions between the two probes, only one of these probes was retained for further evaluation - Xp22. 1p31 was excluded from subsequent assessment.

It is necessary to note that in our study, 3q26 was found to be excellent at detecting both cancer and high-grade lesions (CIN2/3), however, it did not meet our criteria established for CIN1 cases that required to detect copy gains in ≤ 10% of samples (3q26 detected gains in 30% of CIN1 cases). However, due to the frequency of implication of 3q26 in disease progression from this assessment as well as published data [24-26], this probe was retained for subsequent probe evaluation studies. Additionally, CEP3 was also added to monitor amplification and deletion for the aforementioned locus.

Copy losses were also evaluated in order to determine their significance in progression of cervical dysplasia. The data from the 35 probes was analyzed based on percentage of samples with copy loss observed in cancer (Table 1). Only one probe, 3p14, was able to detect copy losses in 80% of cancer samples. 7 probes were only able to detect copy losses in 10-20% in the same set of samples. In detecting CIN2/3 lesions, 3p14 was able to detect copy losses in 20% of these samples and 4 other probes were able to detect copy losses in 10-11% of cases (see Table 1). Results show that in the CIN1 category the percent of cases showing copy number loss varied from 0 to 22% for the tested probes (Table 1). Therefore, only 3p14 was retained for subsequent investigation in a larger sample cohort.

### Evaluation of probes based on mean signals per cell

**Figure 1:**
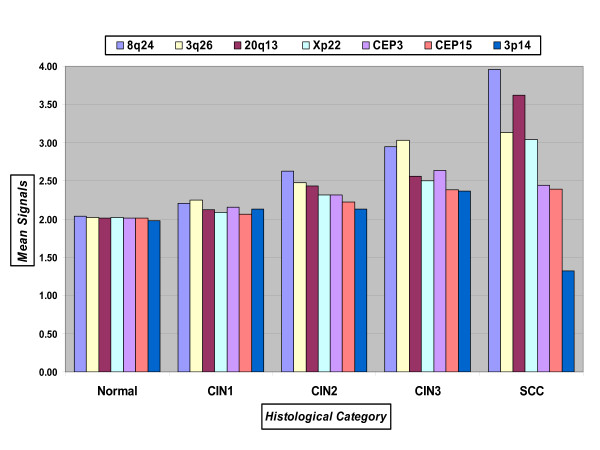
**Mean number signals per specimen for each probe across all histological categories**.

In order to select the best probes for detecting high-grade disease we compared probe gains and losses for 8q24, CEP15, Xp22, 20q13, 3p14, 3q26, and CEP3 on a larger set of biopsy specimens. Enumeration data from 106 specimens was analyzed according to the mean number of signals per cell for each histological category evaluated: Normal (n = 21), CIN1 (n = 19), CIN2 (n = 27), CIN3 (n = 20), and SCC (n = 19). The data is summarized in Figure 1. As expected, the mean of signals for specimens diagnosed as Normal was approximately 2, ranging from 1.98 to 2.04. For CIN1 samples the mean of signals showed more variation among specimens, however was still relatively close to 2. The lowest observed mean for CIN1 samples was 2.06 (CEP15) followed by 2.09 (Xp22). Subsequently, 20q13 had a mean of 2.12 signals, and 3p14 and CEP3 had a mean of 2.13 and 2.16 signals, respectively. The two probes having the highest mean of signals for the CIN1 category were 8q24 with 2.21 signals and 3q26 with 2.25 signals. The CIN2 category revealed a much larger discrepancy between the candidate probes. 3p14 and CEP15 had a mean of signals of 2.13 and 2.22, respectively, whereas, 20q13, 3q26, and 8q24 revealed probe signal means of 2.43, 2.48, and 2.63, respectively. CEP3 and Xp22 were both found to have a mean of 2.32 signals. Further stratification of probes was seen in the CIN3 specimens. The two probes with the highest mean for copy numbers were 8q24 and 3q26 (2.95 and 3.03, respectively). The next two probes showed an appreciably lower mean: CEP3 with 2.64 average copies and 20q13 with 2.56 average copies. The remaining four probes had average copy numbers of 2.50, 2.38, and 2.37 (Xp22, CEP15, and 3p14, respectively).

In cancer cases, 3q26, 8q24, 20q13 and Xp22 revealed an increase in mean copy number. CEP15 average copy number did not change. However, a decrease in copy number was observed for CEP3 and more importantly, a noteworthy decrease observed for 3p14. The mean copy number for this probe was found to be 1.32 thereby revealing copy losses as opposed to gain in the cancer category, which was expected and is concordant with the previously obtained results.

### Change of mean copy number between categories

**Figure 2:**
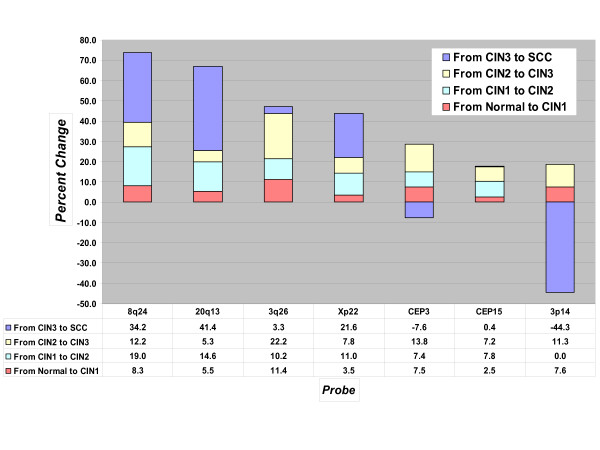
**Percent change of mean probe copy numbers between histological categories**. Values above zero represent positive values (gains) and values below zero represent negative values (losses).

To give further insight into the differences between probes amongst all the histological categories, analysis was performed according to percent change of mean copy number (see Figure 2). Based on the total copy number percent change from Normal to Cancer specimens, 4 probes have shown an increase greater than 40%: 8q24 (73.7%), 20q13 (66.9%), 3q26 (47.1%), and Xp22 (43.8%). The increase in copy gains from the Normal to the CIN1 category was relatively minor with all but one probe (3q26) below 10%. These four probes were also capable of revealing copy gains between CIN1 and CIN2 histological categories with a percent change range of 11.0% to 19.0%. The performance of these four probes varied in high-grade lesions (CIN2-3) and cancer. 3q26 demonstrated the highest percent change from CIN2 to CIN3, 22.2%, followed by 8q24 (12.2%), Xp22 (7.8%), and 20q13 (5.3%). The change from CIN3 to cancer was best detected by 20q13 (41.4%), 8q24 (34.2%), and Xp22 (21.6%). 3q26 showed a lesser ability to detect the CIN3 to cancer progression with a 3.3% change. 3p14 revealed overall copy losses when % total change and % change from CIN3-SCC were evaluated.

### Threshold Determination

**Figure 3:**
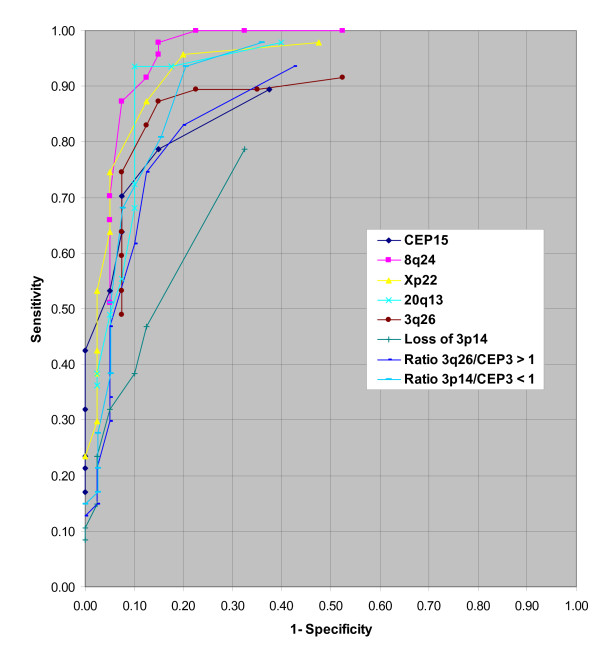
**Receiver Operating Characteristic Curve (ROC) for threshold determination using histological samples**. The ROC analysis utilized 1 to 10 cells (from right to left on the graph, respectively) for curve determination.

Using the data obtained from enumeration, thresholds ranging from 1 to 10 aneusomic cells were evaluated via receiver operating characteristic (ROC) curves to determine the optimum threshold for evaluation of probes. The results from this analysis are displayed in Figure 3. Each probe plot consists of 10 data points which represent the thresholds from 1 to 10 aneusomic cells (from right to left). ROC curves simultaneously evaluate both the sensitivity and specificity of a probe for each threshold. For ROC curve analysis, sensitivity was defined as percent positivity for CIN2 and CIN3 specimens and specificity was defined as percent positivity for Normal and CIN1 specimens. The curves shown in Figure 3 reveal that typically, with thresholds of 1 or 2 cells, sensitivity is high but specificity is low. Conversely, with thresholds ranging from 6 to 10 cells, most probes have high specificities but relatively low sensitivities. The remaining range of thresholds (3-5 cells) exhibit acceptable levels of both sensitivity and specificity for most of the probes being evaluated. Based on the ROC curve results, we selected a 4 cell threshold for further probe analyses.

### % Positivity per Histological Category

The results of the percent positivity analyses using the 4 positive cells threshold are summarized in Table 2. Four probes demonstrated an excellent ability to detect SCC in the sample cohort: 8q24, 3q26, Xp22, and 20q13 (100%, respectively). For the CIN3 category, 8q24 and 3q26 were able to detect 100% and 95% of cases, respectively, while Xp22 and 20q13 performance declined (80% and 70% of cases detected for each probe). 8q24 continued to perform well in detecting CIN2 samples with a positivity rate of 96.3%; concurrently, 3q26 had a positivity rate of 81.5%. The positivity rate continued to decrease with 20q13 and Xp22 probes with 74.1% and 70.4%, respectively. CEP15 revealed the lowest level of performance among all the probes for both the high-grade and cancer categories, including the respective combined categories.

**Table 2:** Percent positivity analyses for each probe across all histological categories (Normal - n = 21; CIN1 - n = 19; CIN2 - n = 27; CIN3 - n = 20; SCC - n = 19)

Threshold = 4 Cells	% Positive cases Normal	% Positive cases CIN^a^1	% Positive cases CIN^a^2	% Positive cases CIN^a^3	% Positive cases SCC^b^
8q24 gain	4.8	26.3	96.3	100.0	100.0

3q26 gain	0.0	31.6	81.5	95.0	100.0

Xp22 gain	0.0	10.5	70.4	80.0	100.0

20q13 gain	0.0	21.1	74.1	70.0	100.0

CEP^c^3 gain	0.0	15.8	66.7	75.0	68.4

CEP^c^15 gain	4.8	10.5	63.0	65.0	68.4

Ratio 3q26/CEP^c^3 > 1	0.0	21.1	51.9	75.0	89.5

Ratio 3p14/CEP^c^3 < 1	9.5	5.6	63.0	75.0	100.0

3p14 < 1 loss	9.5	0.0	33.3	30.0	89.5

^a ^= cervical intraepithelial neoplasia

^b ^= squamous cell carcinoma

^c ^= centromeric probe

A low percent positivity rate for Normal and low-grade cervical lesions (CIN1) was also an important factor for probe evaluation since low values indicate good specificity. For the Normal category, the percent positivity rate for all probes was low ranging from 0% to 4.8% (see Table 2). The percent positive rate for the CIN1 category displayed a higher diversity amongst the probes, ranging from 10.5% (Xp22 & CEP15) to 31.6% (3q26).

Percent positivity analysis was also performed for probes ratio using CEP3 to analyze amplification of the chromosome arm relative to the respective chromosome (Table 2). The ratio of 3q26 gain/CEP3 gain ≥ 1 was negative for Normal specimens (0%) and displayed relatively low positivity for CIN1 specimens (21.1%). The performance was marginal for CIN2 samples (51.9% positivity) and improved for the CIN3 and SCC categories (75% and 89.5% positivity). Although an improvement was seen in evaluating the CIN3 and SCC categories, the ratio of 3q26 to CEP3 did not exceed the individual performance of 3q26.

Since 3p14 displayed a higher frequency of losses in cancer cases combined with a relatively low frequency of gains in high-grade dysplasia, the percent positivity of samples with copy loss was evaluated. Percent positivity (using loss rather than gain) for the Normal and CIN1 categories was found to be low (9.5% and 0%, respectively), indicating good specificity for 3p14 probe. In CIN2 and CIN3 specimens percent positivity was still low (33.3% and 30.0%). A dramatic increase was observed for percent positivity in the SCC category (89.5%) using 3p14 losses as the criterion. The overall percent positivity rates for high-grade and high-grade/cancer specimens analyzed with 3p14 loss were 31.7% and 50.9%. Based on these moderate positivity rates, 3p14 was determined to be an inadequate probe for detecting high-grade dysplasia/cancer and was excluded from subsequent analysis.

The ratio of 3p14 loss to CEP3 revealed some differences in performance when compared to 3p14 loss alone (see Table 2). Percent positivity for Normal cases was the same for both 3p14 loss alone and the 3p14 loss/CEP3 ratio (9.5%), however, 3p14 loss did not detect any CIN1 cases whereas the 3p14 loss/CEP3 ratio detected a few (5.6%). However, performance of the 3p14 loss/CEP3 ratio versus 3p14 loss was vastly improved for CIN2 and CIN3 categories (63.0% and 75.0% versus 33.3% and 30.0%, respectively). For the SCC specimens, the 3p14 loss/CEP3 ratio also showed improvement over 3p14 loss alone (100% vs. 89.5%).

Overall, based on the histological analyses, 8q24 and 3q26 were determined to show the highest degree of copy number changes as measured by the mean value amongst all the probes. They showed best sensitivity to copy number changes from CIN2 to CIN3 categories and from CIN3 to Cancer categories. And finally, they had the best performance in identifying high-grade lesions and cancer as well as the ability to distinguish low-grade and high-grade lesions.

### Cytological Analysis of 8q24 and 3q26 probe performance

**Figure 4:**
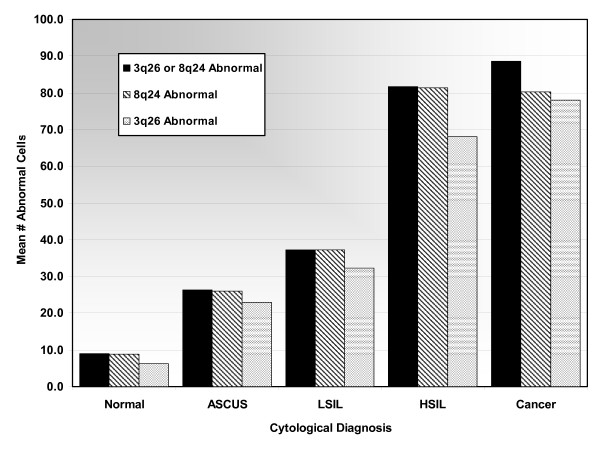
**Mean number of abnormal cells for each cytological category using 3q26 or 8q24, 8q24, and 3q26**. The 3q26 or 8q24 Abnormal column (black) represents all cells found positive for either 3q26 or 8q24, the 8q24 Abnormal column (diagonal stripes) represents all cells found positive with 8q24, and the 3q26 Abnormal column represents all cells that were found positive by 3q26.

Based on the probe performance shown for histological samples, 8q24 and 3q26 probes were formulated into one probe set for testing and assessing their utility for cytology specimens. 118 cytological specimens were enumerated and the data was analyzed according to mean number of abnormal cells per cytological category for 8q24, 3q26, and 8q24 or 3q26 combined (see Figure 4). The numbers of abnormal cells detected by 8q24, 3q26, and their combination correlated with increasing grades of dysplasia. As presented in Figure 4, both probes exhibited good performance in detecting abnormal cells for all cytological categories, although some slight quantitative differences between the probes were observed, particularly in HSIL samples. The use of either 8q24 or 3q26 in combination to determine cellular abnormality was found to be the best option for all cytological categories, further confirming a complementary effect of these probes. Based on these results using the criterion of either 8q24 or 3q26 copy gains to determine cell abnormality would provide the best overall capability in detecting aneusomy.

In order to determine the sensitivity and specificity of using the aforementioned criterion, ROC curves were utilized similarly to our previous analysis of histological samples. NIL, ASCUS, and LSIL samples were used to represent specificity and HSIL and Cancer samples were used to determine sensitivity. The enumeration data was evaluated at threshold values of 1, 5, 10, 15, 20, 25, 30, 35, 40, 45, and 50 abnormal cells. The ROC curve revealed an inflection point at the 30 cell threshold threshold, indicating the best overall performance for sensitivity and specificity (data not shown). Using the 30 cell threshold, sensitivity and specificity were found to be 92.3% and 81.0%, respectively. These results clearly indicate a potential of a FISH based assay for detecting abnormal cells in cervical cytology specimens.

## Discussion

In this study we described the assessment of FISH probes that were found to be highly associated with progression of cervical dysplasia. The initial selection process evaluated the frequency of gains and losses of 35 probes of centromeres and specific loci that were identified in literature as having some implication in cervical disease [9-23]. Preliminary evaluation focused on the highest frequencies of gains/losses in the SCC category, followed by CIN2/3, combined with the lowest frequency of gains and losses in CIN1. The result of this analysis yielded a smaller subset of probes: 8q24, 20q13, CEP15, and Xp22. 3q26 also exhibited good performance for high-grade and cancer cases and was further evaluated in the larger set of biopsy samples. Only one probe - 3p14 - revealed substantial losses that, in combination with previously published data [13,19,21] warranted its retainment for further testing.

Our subsequent comprehensive evaluation distinguished 2 probes that exhibited the highest correlation with high-grade lesions, 8q24 and 3q26. These two probes had the highest signal means for CIN2 and CIN3 cases, respectively (Figure 2). Figure 3 illustrates the overall signal mean changes between categories and shows that the combination of 8q24 and 3q26 offers comprehensive coverage from CIN2 to SCC. 8q24 excels in detecting CIN2 and SCC, whereas 3q26 outperforms all other probes in detecting CIN3 lesions. This is consistent with several reports where 3q26 locus was demonstrated to be frequently associated with cervical dysplasia and transition to invasive cancer [24-27].

Another probe that displayed good performance in SCC as well as CIN2 samples, as evidenced by signal mean and % signal mean change was 20q13. This chromosomal region comprises several important genes, including Aurora kinase A, which was implicated in the development of aneuploidy through deregulation of centrosome formation [28-30]. Increased copy number of 20q13 region was shown to be associated with both squamous cell carcinoma and adenomacarcinoma of the cervix [22]. However, this probe was not as effective at detecting CIN3 samples as 3q26, which are of critical importance in cervical disease progression, predominantly for underlying lesions not discovered through cytological analysis. Additionally, when compared to 8q24, 20q13 performance, although similar, was found to be consistently lower in overall signal mean as well as percent change of means from low to high-grade categories.

CEP15 was the only centromeric probe that passed the initial criteria for selection and thus was used for further testing. This probe clearly did not perform as well as perceived based on the initial screening. The change in overall signal mean for CEP15 across all categories was limited (2.01 to 2.39, Normal to SCC) and the percent change was less than that of the aforementioned probes. The poor performance of centromeric probes when compared with locus specific arm probes perhaps indicates that copy number changes in the chromosomal arm and bands occur earlier than whole genome polyploidisation.

The disparity in performance among the probes was clearly evident in percent positivity analysis for histology samples (Table 2). 8q24 and 3q26 were the most valuable probes for detecting high-grade (CIN2/3) and SCC lesions. Using a 4 cell threshold, 8q24 detected 98.8% of high-grade and cancer cases followed by 3q26 with 92.2%. Among the other probes tested, Xp22 and 20q13 performance in high-grade and cancer cases was similar.

Deletions of several genetic loci have previously been implicated in cervical dysplasia and cancer. The frequency of copy loss, as evidenced in our study, was shown to have a minimal correlation with the high-grade categories of dysplasia. For instance, 3p14 probe loss was observed in 33.3% of CIN2 samples and 30.0% of CIN3 samples, whereas 89.5% of SCC samples displayed a 3p14 deletion. The poor performance of 3p14 in high-grade dysplasia and excellent performance in cancer leads us to believe that the loss of 3p14 locus, which comprises the FHIT gene, is a late event in cervical disease [19].

The positivity rate for CIN1 specimens using 8q24 and 3q26 probes was found to be 15.5% and 15.8%, respectively; it was significantly lower for Normal cases (4.8% and 0%, respectively) indicating good specificity. It is possible that CIN1 cases determined to be positive by 8q24 and 3q26 may in fact be harboring molecular changes indicative of disease progression that were not visualized during histological analysis. In fact, several studies have demonstrated that a proportion of patients with ASCUS and LSIL may progress to higher grade of dysplasia and even cancer [31-33]. It is therefore feasible that 8q24 and 3q26 may have an advantage of earlier detection of severe dysplasia when compared to the other probes evaluated as evidenced by percent positivity rates of 20q13 and Xp22 for the CIN1 category (Table 2). Indeed, 3q26 has already been shown to have prognostic value in identifying a high-progression risk in low-grade lesions [26]. It is possible that the increased sensitivity of 8q24 and 3q26 probes may provide further stratification and earlier identification of increased dysplasia in CIN1 patients.

The application of 8q24 and 3q26 probes to cytological samples revealed a strong correlation between aneusomy and increasing cytological diagnosis. The ability of these probes to detect copy gains in ASCUS and LSIL samples is significant in that molecular changes possibly representing underlying high-grade dysplasia can be discovered without the use of invasive procedures. The use of 3q26 as persistence-progression indicator has previously been shown [34] and may aid in avoiding unnecessary colposcopies and biopsies, particularly in women who do not present obvious high-grade dysplasia. Moreover, the use of 3q26 has been shown to accurately distinguish between HSIL and LSIL [35]. The inclusion of 8q24 in our study for cervical screening has shown an increase in both sensitivity and specificity when compared to using either 8q24 or 3q26 alone for ASCUS, LSIL, and HSIL specimens. In normal specimens both probes identified cells with low levels of abnormality, which can be attributed to several factors including inflammatory/reactive changes and cells in the process of division, presenting tetrasomic signal counts. Although these cells were detected utilizing 8q24 and 3q26, a cutoff of 30 abnormal cells prevented these cells from impacting the specificity of these probes. Therefore, our findings in cytological samples are in accord with our histological results and further support the utility of 8q24 and 3q26 as important markers in detecting cervical disease.

## Conclusions

It is clear that several genes are involved in cervical dysplasia and cancer [36]. In our study, we identified that gene amplification is much more frequently encountered in cervical dysplasia compared to gene loss. Only one probe, 3p14, exhibited significant losses, which were mainly detected in cancer cases. Conversely, several probes demonstrated substantial rates of amplification in high-grade dysplasia and cancer. However, based on all the analyses presented, 8q24 and 3q26 clearly outperform the other probes tested in detecting high-grade dysplasia and cancer. Moreover, 8q24 and 3q26 probes may have a utility as prognostic markers in cervical dysplasia, particularly in underlying high-grade lesions. Additional studies need to be performed to establish a greater confidence in the clinical utility of 8q24 and 3q26 FISH probes as prognostic markers.

## Competing Interests

The authors declare that they have no competing interests.

## Authors' contributions

FP analyzed the cytological samples, performed the statistical analysis, and drafted the manuscript. MS analyzed the histological and cytological samples. SS analyzed the histological and cytological samples. AO analyzed the histological and cytological samples and assisted in selection of probes. RA selected appropriate samples and generated both cytological and histological diagnoses for the specimens and assisted in the study design and coordination. CM selected appropriate samples and generated both cytological and histological diagnoses for the specimens and assisted in the study design and coordination. LM assisted in the study design and coordination and helped draft the manuscript. WK conceived the study and participated in the study design. IS conceived the study, coordinated the study and its design, and helped draft the manuscript. All authors read and approved the final manuscript.

## Pre-publication history

The pre-publication history for this paper can be accessed here:

http://www.biomedcentral.com/1471-2407/10/432/prepub
